# Enhanced surveillance for the Third United Nations Conference on Small Island Developing States, Apia, Samoa, September 2014

**DOI:** 10.5365/WPSAR.2016.7.4.002

**Published:** 2017-02-06

**Authors:** Paul White, Salanieta Saketa, Alexis Durand, Saine Vaai-Nielsen, Tile Ah Leong-Lui, Take Naseri, Ailuai Matalima, Filipina Amosa, Alize Mercier, Christelle Lepers, Vjesh Lal, Richard Wojcik, Sheri Lewis, Adam Roth, Yvan Souares, Onofre Edwin Merilles, Damian Hoy

**Affiliations:** a Epidemiology and Laboratory Capacity Programme, Public Health and Hospital Emergency Preparedness Office, Commonwealth Health Care Corporation, Saipan, Commonwealth of the Northern Mariana Islands.; b Research Evidence and Information Programme, Public Health Division, Pacific Community.; c Notifiable Disease and Surveillance and International Health Regulations Division, Ministry of Health, Samoa.; d National Laboratory, Tupua Tamasesse Meaole Hospital, Samoa National Health Services.; e Johns Hopkins University Applied Physic Laboratory.

## Abstract

The Ministry of Health in Samoa, in partnership with the Pacific Community, successfully implemented enhanced surveillance for the high-profile Third United Nations Conference on Small Island Developing States held concurrently with the popular local Teuila festival during a widespread chikungunya outbreak in September 2014.

Samoa’s weekly syndromic surveillance system was expanded to 12 syndromes and 10 sentinel sites from four syndromes and seven sentinel sites; sites included the national hospital, four private health clinics and three national health service clinics. Daily situation reports were produced and were disseminated through PacNet (the e-mail alert and communication tool of the Pacific Public Health Surveillance Network) together with daily prioritized line lists of syndrome activity to facilitate rapid response and investigation by the Samoan EpiNet team. Standard operating procedures for surveillance and response were introduced, together with a sustainability plan, including a monitoring and evaluation framework, to facilitate the transition of the mass gathering surveillance improvements to routine surveillance.

The enhanced surveillance performed well, providing vital disease early warning and health security assurance. A total of 2386 encounters and 708 syndrome cases were reported. Influenza-like illness was the most frequently seen syndrome (17%). No new infectious disease outbreaks were recorded. The experience emphasized: (1) the need for a long lead time to pilot the surveillance enhancements and to maximize their sustainability; (2) the importance of good communication between key stakeholders; and (3) having sufficient staff dedicated to both surveillance and response.

## Introduction

The Third United Nations Conference on Small Island Developing States (SIDS) was held in Apia, Samoa, from 1 to 4 September 2014. Attracting over 3000 delegates from more than 100 countries and territories, (*1*) this was the largest international event ever hosted by Samoa – a Pacific island nation of 187 820 people. (*2*) The SIDS conference occurred simultaneously with the annual Teuila festival, one of the Pacific region’s largest cultural events.

Large gatherings present considerable public health disease risks, (*3*, *4*) particularly where there is a large and diverse international population influx. This was demonstrated in Samoa, as the two events coincided with outbreaks of chikungunya (CHIKV) locally (*5*) and with the largest ever Ebola virus disease (EVD) outbreak in West Africa. While the EVD importation risk to Pacific island countries and areas was low, (*6*) the stress on the Samoan health system to accommodate EVD cases in the event of any incidences would have been very high. The evolving CHIKV outbreak and ongoing dengue fever, measles and conjunctivitis outbreaks in neighbouring Pacific island countries and areas (*5*) could have overwhelmed local health resources and disrupted the SIDS conference.

As part of meeting health security preparations for the SIDS conference, including International Health Regulations (2005) requirements for improving surveillance, the Samoan Ministry of Health (MoH) asked the Pacific Community (SPC) for technical support in planning, implementing and managing enhanced surveillance for the event. Enhanced surveillance is a practical response to elevated public health risks arising from “events attended by a sufficient number of people to strain the planning and response resources of a community state or nation.” (*7*) As a foundation of disease prevention and control, (*8*) surveillance provides early warning of potential disease outbreaks, allowing timely response and prioritized management of surge demands on health services. Mass gathering surveillance is commonly implemented in many countries for a range of sporting, (*4*, *5*) religious and cultural festivals, (*3*, *9*) and international political meetings, (*10*) ranging in size from a few thousand people (8th Micronesian Games) to millions (Hajj pilgrimages).

SPC has accumulated considerable Pacific experience in implementing enhanced surveillance during mass gatherings, including the 2012 11th Festival of Pacific Arts, Solomon Islands; the 2013 Pacific Mini-games, Wallis and Futuna; and the 2014 8th Micronesian Games, Pohnpei State, Federated States of Micronesia. Here we describe the SIDS conference surveillance implemented by the Samoa MoH and SPC, highlighting lessons that may be helpful to public health planners in preparation for disease surveillance for mass gatherings.

### Purpose of the mass gathering enhanced surveillance system

There were three primary purposes for the enhanced surveillance: (1) to provide a simple surveillance system for rapidly detecting and responding to disease episodes or outbreaks in a timely and effective manner; (2) to disseminate strategic epidemiological information throughout the Pacific region; and (3) to sustainably improve disease surveillance in Samoa beyond the mass gathering event.

### Planning and implementation of the enhanced surveillance

**Fig. 1:**
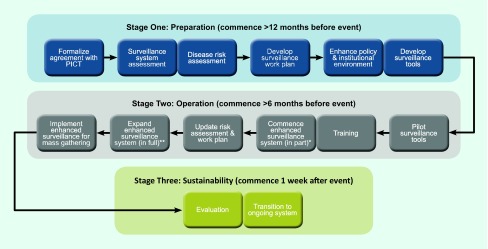
SPC process map of the steps for the implementation of mass gathering surveillance

SPC employs a three-stage process for enhanced surveillance (see **Fig. 1**) comprising preparation, operation and sustainability functions. Preparation should commence 12 months before the event and includes assessing the surveillance system and disease risk and developing a work plan for enhanced surveillance. Surveillance operations of the second phase commences up to six months ahead of the event and includes pilot testing, training and implementing the enhanced surveillance system. The sustainability phase starts one week after the event and involves transition to the regular surveillance system and evaluation of the impact of the enhanced surveillance.

* Expand to all new sentinel sites; continue with weekly reporting; commence use of web-based data entry and analysis

** Change from weekly reporting to daily reporting

#### Stage 1 – Preparation: surveillance needs and disease risk assessment

The surveillance needs for the SIDS conference were determined by assessing: (1) the current scope and scale of the existing surveillance system; (2) the number and geographical diversity of SIDS conference delegates; and (3) the disease risks.

Four themes emerged from the health risk assessment: the current CHIKV outbreak, outbreaks of other infectious diseases in Pacific island countries and areas (dengue, measles), fear of EVD importation and the increased pressure on existing health services if an outbreak occurred. Based on the assessment and building on the existing syndromic surveillance system, the following modifications were made for the mass gathering surveillance: (*11*)

- changing reporting frequency from weekly to daily;
- increasing the number of syndromes reported from seven to 12 (**Table 1**) covering a wide spectrum of disease priorities, including national and regional outbreaks, severe and notifiable diseases and food- and waterborne diseases;

**Table 1:** SIDS conference enhanced surveillance syndromes and case definitions

Syndromes	Case definitions	Important diseases to consider
Acute fever and rash	Sudden onset of fever (> 38 °C) AND acute non-blistering rash	Measles, dengue fever, rubella, meningitis, leptospirosis, chikungunya
Watery diarrhoea	3 or more watery stools in 24 hours	Cholera
Non-watery diarrhoea	3 or more loose stools in 24 hours	Viral or bacterial gastroenteritis, including food poisoning and ciguatera fish poisoning
Influenza-like illness	Sudden onset of fever (> 38 °C) AND cough or sore throat	Influenza, other viral or bacterial respiratory infections
Prolonged fever	Any fever (> 38 °C) lasting 3 or more days	Typhoid fever, dengue fever, leptospirosis, malaria
Chikungunya-like illness	Sudden onset of fever PLUS pain in multiple joints EITHER with or without rash	Chikungunya
Dengue-like illness	Fever for at least 2 days PLUS at least two of the following: nausea or vomiting, muscle or joint pain, severe headache or pain behind the eyes, rash, bleeding	Dengue fever, dengue haemorrhagic fever, dengue shock syndrome
Acute flaccid paralysis	Any cases of acute flaccid paralysis in a child < 15 years old or Guillain-Barré syndrome or suspected polio in any age	Acute poliomyelitis
Neonatal tetanus	Any neonate with a normal ability to suck and cry during the first 2 days of life, and between 3 and 28 days of age cannot suck and cry normally and becomes stiff or has convulsions or both	Neonatal tetanus
Fever and jaundice	Any fever (> 38 °C) AND jaundice	Hepatitis A
Acute fever and neurological symptoms	Sudden onset of fever with neurological symptoms, altered mental state, confusion, delirium, disorientation, seizure	Meningococcal meningitis, viral meningitis, other viral encephalitis (e.g. West Nile virus)
Foodborne diseases	Clustering of at least 2 cases having gastro-intestinal symptoms originating from same food outlet or catering site	Includes salmonella, staphylococcus, clostridium, campylobacter and rotavirus infections
- increasing the number of reporting sentinel sites in Apia from one to 10 to achieve greater population coverage;
- providing prioritized daily case reports of syndrome activity to facilitate rapid response and investigation; and
- introducing and adapting the Suite for Automated Global Electronic bioSurveillance Open ESSENCE (SAGES OE) surveillance system for data storage and analysis.

#### Stage 2 – Operation: implementation of the enhanced surveillance

A two-day training course was held for the sentinel site focal points with refresher training occurring during daily data collection rounds. Training focused on:

- understanding the syndrome case definitions;
- accurate completion of the surveillance register; and
- specimen collection and referral of laboratory samples.

The surveillance was tested in the week preceding the SIDS conference and became operational on 26 August. The enhanced surveillance continued until 19 September, and the daily reporting ended on 6 September.

### Data collection

A surveillance register system captured daily acute care encounters and syndrome cases. The surveillance registers were collected at each sentinel site each day and exchanged for new registers.

### Surveillance tools, data analysis and generation of situation reports

SAGES OE is a freeware tool designed by Johns Hopkins University Applied Physics Laboratory (JHU-APL). (*12*) SAGES OE was adapted for the enhanced surveillance by JHU-APL and SPC and had successfully been used previously by SPC for mass gathering surveillance; (*13*) however, technical challenges in locally hosting the system precluded the full use of SAGES OE at the SIDS conference, so a spreadsheet-based alternative was used to store the daily data and generate graphical output. This output was incorporated into daily situation reports (SitReps), providing descriptive summaries (including laboratory results) and narrative interpretation of daily syndrome and encounter activity.

### Laboratory surveillance

A laboratory surveillance focal point was selected to link syndromic surveillance and laboratory surveillance at the national laboratory in the Tupua Tamasese Meaole Hospital (TTMH). The diagnostic process included off-island sample referral protocols for confirmatory testing for epidemic-potential diseases.

### Information exchange, investigation and response

The surveillance team provided early warning alerts for immediate response follow-up of any prioritized syndrome cases (such as acute fever and rash or bloody diarrhoea) that were found at the time of daily data collection. Additionally, daily case reports were given to the response team for follow-up investigation. SitReps were e-mailed to the MoH and the SIDS organizing stakeholders and were disseminated to regional public health professionals via the PacNet Pacific regional public health e-mail network.

#### Stage 3 – Transition, sustainability, and monitoring and evaluation

A sustainability plan was generated to transition improvements from the mass gathering surveillance to the routine surveillance system to harness the considerable effort involved in implementing the enhanced surveillance. This included a monitoring and evaluation plan to benchmark surveillance performance for future assessment. The sustainability plan was discussed during a joint SPC and MoH debriefing session at the end of the mass gathering.

## Results

A total of 2386 encounters were seen at the 10 sentinel sites, from 26 August to 6 September 2014. Daily encounters at the sentinel sites ranged from 0 to 299. Seven hundred eight encounters (30%) presented with syndromes under surveillance (see **Table 2**). Three syndromes accounted for nearly 90% of all syndrome cases (*n* = 631) and more than a quarter of all encounters (26.4%): influenza-like illness – nearly 60% of syndrome cases (*n* = 402), acute fever and rash – 19% (*n* = 134) and chikungunya-like illness – 13% (*n* = 95). No acute flaccid paralysis, neonatal tetanus or foodborne diseases were reported. One case of dengue-like illness was investigated and tested positive by rapid test (NS1, Bio-Rad Laboratories, Marnes-la-Coquette, France), with evidence of acute (probable primary) dengue fever infection. (*13*) Most syndrome cases were reported among Samoan nationals, and no importation of any infectious diseases among delegates and visitors were reported.

**Table 2:** Reports of syndrome cases by all points of care: 26 August to 6 September 2014

Syndrome	Number of syndrome cases	Syndrome cases as a percentage of all encounters	Syndrome cases as a percentage of all syndromes
Influenza-like illness	402	16.8	56.8
Acute fever and rash	134	5.6	18.9
Chikungunya-like illness	95	4.0	13.4
Watery diarrhoea	23	1.0	3.2
Prolonged fever	17	0.7	2.4
Non-watery diarrhoea	16	0.7	2.3
Dengue-like illness	15	0.6	2.1
Fever and neurological symptoms	4	0.2	0.6
Fever and jaundice	2	0.1	0.3
Acute flaccid paralysis	0	0	0
Neonatal tetanus	0	0	0
Foodborne disease outbreak	0	0	0
Total syndrome cases	708	29.7	100
**Total acute encounters**	**2386**	-	-

## Discussion

No new infectious disease outbreaks were recorded for the SIDS conference, and the surveillance system performed well, providing important assurances for public health safety. The CHIKV outbreak was well managed and did not impact the conference. Increasing reporting frequency from weekly to daily, increasing the number of syndromes and the number of sentinel sites improved public awareness of the health risks to the local and international community. These measures together with sentinel clinicians’ awareness and accurate identification of syndrome definitions improved surveillance sensitivity. This is shown with 30% of encounters as syndrome cases, compared to only 7–10% of encounters recorded as syndrome cases in previous SPC-implemented mass gathering surveillance activities in the Pacific. (White P, Mercier A, Saketa S, Hoy D. Sustaining Enhanced Syndromic Surveillance in Pohnpei (FSM). Noumea: The Pacific Community (SPC), unpublished report. 2014), (Dr Sala Saketa, The Pacific Community (SPC), personal communication, 12 January 2014)

The benefits of enhanced surveillance can be sustained when the mass gathering surveillance experience is integrated into long-term surveillance improvement plans rather than being treated as an isolated activity occurring only during a discrete time frame. Similarly, it is more likely that the extra effort involved in mass gathering enhanced surveillance will be implemented when the work involved is similar to the usual surveillance. The SIDS conference enhanced surveillance was implemented by building on the existing weekly surveillance, facilitating straightforward transition after the conference as well as enabling lessons learnt and benefits gained to be readily applied.

Lessons learnt from the SIDS conference enhanced surveillance experience identified important points for the future planning of mass gathering surveillance:

Early preparation is essential, avoiding the temptation to leave surveillance implementation to the ‘last minute’. Planning for the enhanced surveillance should start at least 12 months before the event. The lead time is necessary to accommodate the preparatory activities in stage 1 and to ensure the operational tasks in stage 2 can be implemented satisfactorily.

Lead time enables planners to embed and pilot the enhanced surveillance, thereby avoiding disruption and time losses during the intense period of surveillance operation and ensuring that newly implemented changes are understood. This was demonstrated at the SIDS conference, where insufficient time was allocated for testing the SAGES OE installation. These technical challenges did not adversely impact the surveillance because a functional substitute was straightforward to use, but this issue illustrated that greater time should have been planned for this activity. As not all increases in disease counts warrant investigation, lead time is also needed to generate and understand baselines arising from increasing the number of reporting sentinels. This frequently occurs where the increase in surveillance coverage results in apparent peaks and troughs in the data resulting from weekend and non-uniform daily operation of sentinel sites (particularly the variable operating times of general practitioners).

It is essential to run a pilot to test the surveillance system before it becomes operational to ensure that the system can perform as expected. Mass gathering surveillance is typified by a short period of intense activity to collect, collate and analyse data and generate meaningful interpretations on a daily basis. The SIDS conference surveillance data collection was time consuming as it relied on visiting each sentinel site daily. This was compounded by the number and locations of the sentinels that more than doubled for the enhanced surveillance from four to 10 and included the international airport 33 km from Apia. The pilot operation was valuable in highlighting the need to increase the number of data collection teams from two to three, to ensure the timely generation and dissemination of SitReps. While running three teams was more labour (and resource) intensive than running two teams, this approach ensured that the daily SitRep could be completed on time every day.

## Conclusions

The enhanced surveillance for the SIDS conference was a large surveillance operation that provided important public health security assurance in support of a high-profile United Nations meeting simultaneously with an equally large local festival that both occurred concurrently with a widespread CHIKV outbreak. Sustainable benefits of the enhanced surveillance included fostering a closer working relationship between public health authorities, the TTMH laboratory and clinical services and improving surveillance activities.

Mass gathering surveillance typically involves a short period of intense activity that can be an extra burden on over-stretched public health resources. However, impacts on resources and staff can be minimized by building on and enhancing existing surveillance activities. This allows for the efficient commencement of enhanced surveillance and transition back to routine surveillance. This approach can result in improvements to public health systems in both capacity (training of staff) and capability (efficiency and quality improvements in the functioning of the surveillance system) that remain long after the mass gathering is over. The benefits from these improvements include better health security arising from the ongoing surveillance operations and indirect benefits from improvements to the epidemiological evidence base available to health planners that accrue through having better-trained surveillance staff, providing better-informed information, from improved data collection and surveillance coverage. Accordingly, mass gathering surveillance can stimulate improvements in public health surveillance that may not have otherwise occurred. The diligent work of the Samoan public health communicable disease surveillance team during the SIDS conference, and the experience they gained in enhanced surveillance, was applied during the mass gathering surveillance for 2015 Commonwealth Youth Games, which was also held in Apia, Samoa.

## References

[1] SIDS – Sustainable Knowledge Platform: Third International Conference on SIDS (http://www.sids2014.org/, accessed 20 March 2015).

[2] Area I. Commonwealth of the Northern Mariana Islands. Saipan: CNMI Department of Commerce, Central Statistics Division; 2010. [cited 2017 January 27]. Available from: http://www.census.gov/population/www/cen2010/island_area/cnmi.html

[3] Thackway S, Churches T, Fizzell J, Muscatello D, Armstrong P. Should cities hosting mass gatherings invest in public health surveillance and planning? Reflections from a decade of mass gatherings in Sydney, Australia. BMC Public Health. 2009 9 08;9(1):324. 10.1186/1471-2458-9-324 19735577 PMC2754454

[4] Kaiser R, Coulombier D. Epidemic intelligence during mass gatherings. Euro Surveill. 2006 12 21;11(12):E061221.3. 17213571 10.2807/esw.11.51.03100-en

[5] SPC Epidemic and emerging disease alerts in the Pacific region interactive map. Noumea: Pacific Community (SPC); 2014 (http://www.spc.int/phd/epidemics/, accessed 27 January 2017).

[6] Ebola virus disease: Risk assessment in the Western Pacific Region 09 October 2014. Manila: World Health Organization Regional Office for the Western Pacific; 2014 (http://www.wpro.who.int/outbreaks_emergencies/wpr_ra_ebola_09oct2014.pdf?ua=1, accessed 7 January 2015).

[7] Communicable disease alert and response for mass gatherings: key considerations June 2008. Geneva: World Health Organization; 2008.

[8] M’ikanatha NM, Lynfield R, Julian KG, Van Beneden CA, de Valk H. Infectious disease surveillance: a cornerstone of prevention and control. Infectious disease surveillance. Oxford: Blackwell Publishing; 2007 10.1002/9780470692097

[9] Memish ZA, Stephens GM, Steffen R, Ahmed QA. Emergence of medicine for mass gatherings: lessons from the Hajj. Lancet Infect Dis. 2012 1;12(1):56–65. 10.1016/S1473-3099(11)70337-1 22192130 PMC7185826

[10] Sugishita Y, Ohkusa Y, Sugawara T, Shimatani N, Nadaoka Y, Kamiya N, et al. Enhanced syndrome surveillance for the fourth Japan-China-South Korea Trilateral summit 2011. J Bioterror Biodef. 2013;4(1) 10.4172/2157-2526.1000126

[11] Kool JL, Paterson B, Pavlin BI, Durrheim D, Musto J, Kolbe A. Pacific-wide simplified syndromic surveillance for early warning of outbreaks. Glob Public Health. 2012;7(7):670–81. 10.1080/17441692.2012.699536 22823595 PMC3419547

[12] Feighner BH, Campbell TC, Katz AT, Wojcik RA, Coberly JS, Patel SV, et al. SAGES Overview: Open-source software tools for electronic disease surveillance in resource limited settings. Johns Hopkins APL Tech Dig. 2014;32(4):652–8.

[13] Hoy D, Saketa ST, Maraka RR, Sio A, Wanyeki I, Frison P, et al. Enhanced syndromic surveillance for mass gatherings in the Pacific: a case study of the 11th Festival of Pacific Arts in Solomon Islands, 2012. West Pac Surveill Response. 2016 9 27;7(3):15–20. http://ojs.wpro.who.int/ojs/index.php/wpsar/article/view/422/705 10.5365/wpsar.2016.7.1.004 27766181 PMC5070428

